# Effects of age and weaning conditions on blood indicators of oxidative status in pigs

**DOI:** 10.1371/journal.pone.0178487

**Published:** 2017-05-24

**Authors:** Arnaud Buchet, Catherine Belloc, Mily Leblanc-Maridor, Elodie Merlot

**Affiliations:** 1 PEGASE, Agrocampus Ouest, INRA, Saint-Gilles, France; 2 Cooperl Arc Atlantique, Lamballe, France; 3 BIOEPAR, INRA, ONIRIS, Nantes, France; Nanjing Medical University, CHINA

## Abstract

Weaning is a source of social, nutritional and environmental disorders that challenge piglet health. This study assesses the relevance of using plasma indicators of oxidative status as biomarkers of health around weaning in pigs. Blood antioxidant potential (BAP), hydroperoxides (HPO), oxidative stress index (OSI, e.g. HPO/BAP), vitamin A and E concentrations were investigated in two different trials. Trial A was carried out in an experimental unit to investigate the effects of age (from 12 to 147 days of age), weaning (at 21 or 28 days of age) and management at weaning (in optimal (OC) or deteriorated (DC) conditions) on those parameters. Trial B was performed in 16 commercial pig farms to describe the variability of these indicators on field between 26 and 75 days of age. In trial A, between 12 and 147 days of age, HPO globally increased (P < 0.001), vitamin E concentration decreased (P < 0.001) whereas BAP and vitamin A concentration remained relatively stable (P > 0.1). Vitamins E and A concentrations dropped 5 days after weaning independently of weaning age, weaning conditions and expression of diarrhea (P < 0.001). Twelve days after weaning, whatever the weaning age, HPO and OSI increased in DC compared to OC piglets (P = 0.05 and P < 0.01) and in piglets exhibiting diarrhea compared to those without diarrhea (P < 0.01 and P < 0.001). In DC pigs, BAP was also decreased (P < 0.05) 12 days after weaning. On trial B, plasma concentrations of vitamins A and E decreased and HPO increased 5 and 19 days respectively after weaning (P < 0,001). Contrarily to trial A, BAP values did not drop after weaning. Piglets which had the lowest ADG (Average Daily Gain) after weaning had greater HPO and OSI and lower vitamin A and E concentrations after weaning but also lower vitamin E concentration before weaning (P < 0.05). In conclusion, HPO or OSI seem to be good indicators of health disorders around weaning and plasma concentration of vitamin E before weaning is associated to growth after weaning.

## Introduction

Pig production has to combine reduction of the use of antibiotics, animal health and welfare and profitability. To develop a better awareness of health and welfare of animals in farms, accurate and easy to measure indicators of health are needed. Robust indicators of oxidative status are possible candidates. Oxidative status reflects the equilibrium between pro- and antioxidant molecules in a living organism [1]. Pro-oxidants molecules are mainly free radicals produced by the respiratory chain of mitochondria. Free radicals oxidize lipids, proteins and DNA, for example, into hydroperoxides, and are responsible for cell and tissue injury if produced in excess. The antioxidant system of the organism involves endogenous components such as glutathione, superoxide dismutase or catalase, and exogenous molecules, supplied by food, such as vitamins (A, C, E) and selenium [1,2]. Under certain physiological circumstances such as immune activation, physical exercise or stress, the production of free radicals can exceed the anti-oxidant potential of the organism [1,2], generating oxidative stress. Oxidative status was proposed as an indicator of health for farm animals because the correction of oxidative stress during several infectious diseases improves health of treated animals [3].

Weaning is a health-challenging period for piglets because of nutritional, environmental and social changes, sometimes associated to bad management practices [4–6]. Furthermore, an increase of oxidative products in plasma has been shown during the second month of life of piglets, and thus after weaning [7–10]. However, it is not clear whether this oxidative stress results from weaning itself or other biological processes related to environmental factors or maturation with age. Indeed, the evolution of oxidative status with age has not been described in growing mammals. Furthermore, in commercial farms, weaning can be carried out in variable management conditions which can affect growth of piglets and health, mainly through the expression of diarrhea [11], and the impact of these conditions on oxidative status has not been described.

The aim of this study was to assess the opportunity to use indicators of oxidative status as biomarkers of piglet health in the context of weaning. For this purpose, blood indicators of antioxidant potential of the animals (Blood Antioxidant Potential (BAP), vitamin A and E concentrations, and hydroperoxides (HPO) were measured together with clinical and growth data in two experiments. A study carried out in experimental facilities was first designed to dissociate effects of age, weaning, and management conditions just after weaning on the evolution of oxidative status (trial A). Then, a second trial was performed in 16 commercial farms in order to describe oxidative status evolution around weaning in the field (trial B).

## Material and methods

### Animals and housing

The experiments were approved by the French ministry of research after evaluation by competent ethics committees in animal experimentation (authorizations #2015070815295160 and #CERVO-2016-6-V, from Rennes (trial A) and Nantes (trial B) committees respectively).

#### Trial A: Effects of management conditions at weaning and age on oxidative status

The experiment was conducted in an INRA experimental unit (Saint Gilles, France). Pietrain x (Large White x Landrace) piglets (NUCLEUS lines, France, n = 66) of 12.3 +/- 0.5 days of age (3.8 +/- 0.9 kg), originating from 12 randomly selected litters, were enrolled in this study. In order to dissociate the effects of age from those of weaning on biological measures, six litters were weaned at 21 (W21) and six others at 28 (W28) days of age. To investigate the influence of management conditions at weaning, for each weaning age and each litter, littermates were equally allocated to optimal or deteriorated post-weaning conditions (OC or DC). The deterioration of weaning conditions was based on bad hygiene, abrupt feed transition, increased animal density, heat stress and social stress. In DC, on the day of weaning, 16 entire males and 16 females were exposed to a cold stress at 20°C for 4 hours before being moved to housing pens that were not cleaned and disinfected neither after the preceding batch, nor until the end of the experiment. Each pen hosted 8 piglets (0.20 m^2^/pig) coming from 6 different litters. One week later, 4 animals from each pen were moved to an empty pen and replaced by 4 non-experimental animals. Feed change from starter to weaner feed was done abruptly at 32 days of age. In optimal conditions (OC), 16 entire males and 18 females were moved from farrowing rooms to a post-weaning room previously cleaned, disinfected and heated at 28°C. Each of the 9 pens contained 2–4 piglets (1.2 x 1.3 m, 0.40 m^2^ / piglet) from only two litters. Transition of OC piglets from starter to weaner feed was done progressively on 3 days (from 32 to 35 days of age). At 61 days of age, all the piglets (21.8 +/- 5.1 kg) were transferred to common standard growing facilities until slaughter.

No antibiotic treatments were used during the study. Two OC W21 piglets died the first week after weaning probably from digestive disorders before any possible detection and medication.

#### Trial B: Oxidative status around weaning in commercial farms

The experiment was conducted from January to June 2015 in 16 commercial farms. Farms reared pigs of the same genetic as in Trial A (NUCLEUS lines) and weaned piglets at 28 days of age. In each farm, two days before weaning, 9 sows from different parities, in accordance with the demographic pyramid of the herd, were selected. Two apparently healthy and middle-weight entire males per litter were included in the study (288 piglets in total). Biological samplings and measurements were performed on these selected piglets at 4 visits occurring two days before (d26), and 5, 19 and 47 days after (d33, d47 and d75) the weaning day. Individual and collective medications administered to piglets during the study were recorded by the farmers.

### Measures on animals and biochemical analysis

In trial A, animals were weighed weekly from 12 to 61 days and then at 88, 119 and 147 days of age. Faeces were observed daily from weaning to 61 days of age and qualified as normal or diarrheic. In trial B, animals were weighed at 26, 33 and 47 days, and faeces were observed at 33 and 47 days of age and qualified as normal or diarrheic.

In trial A, blood was collected weekly from all piglets from 12 days of age until one week after weaning, and then at 88, 119 and 147 days of age. From 1 week after weaning to 61 days of age, blood samples were also collected weekly but only on half of the piglets alternatively in order to limit the frequency of blood collection for each animal. Blood samples were collected at jugular vein in 1 Venosafe tube (Terumo, Japan) of 5 mL containing heparin as anticoagulant. In trial B, blood was collected in 10 mL heparinized tubes from the 288 selected piglets at 26, 33, 47 and 75 days of age. Blood was kept on ice until arrival to the laboratory, where it was centrifuged at 3000*g* for 15 minutes at 4°C and plasma was stored at -20°C until analyses.

HPO and BAP of heparin plasma were assayed by analytical methods using commercial kits (dROM and BAP tests, Diacron, Grosseto, Italy) on an automated analyser (Konelab 20, Thermo Electron Corporation). The dROM test measures the concentration of HPO generated by the peroxidation of lipids, proteins or nucleic acids [12]. Briefly, a 2 μL of heparin plasma sample was added to 100 μL of acidic buffer and 1μL of chromogen. Absorbance was read every 98 seconds for 980 seconds at 505 nm. Standard curve with 8 points and internal control sample were used (intra and inter-assay coefficients of variation (CV) of 6 and 8% respectively). The results of the test are expressed in CARRU (Carratelli Unit, 1 CARRU = 0.08 mg H_2_O_2_/100mL of sample).

BAP results from the combined effects of many antioxidants such as uric acid, ascorbic acid, proteins, alpha-tocopherol or bilirubin [13]. Briefly, a 5 μL heparin plasma sample was added to a 210 μL solution of ferric chloride and thiocyanate derivate. Absorbance was read at 505 nm. Standard curve with 8 points and internal control sample were used (Intra and inter-assay CV of 2 and 6%, respectively). The results are expressed in μmol/L of equivalent vitamin C used as an iron-reducing reference agent.

An Oxidative Stress Index (OSI) was calculated as the ratio of HPO to BAP (CARRU/μmol/L of Vit C) as described by Sharma et al. [14].

Vitamin E (alpha-tocopherol) and vitamin A (retinol) concentrations were assayed on heparin plasma samples by liquid chromatography (HPLC) on a dedicated column (Chromsystems, Germany).

### Statistical methods

Analysis of variance were done using the *lmer* function from *lme4* package [15] of R software [16]. The effect of age (from 12 to 147 days of age in trial A, at 26, 33, 47 or 75 days of age in trial B) on growth and blood variables was analyzed in models including age as main effect, and piglet (Trial A) or piglet nested in the farm (Trial B) as random effect. In trial A, to investigate the variations of average daily gain (ADG), weight and blood variables around weaning, only data collected from 9 days before to 19 days after weaning were used and the models included time to weaning (from -9 to +19 days), management conditions (OC vs DC), age at weaning (W21 vs W28) and their interactions as main effects, and piglet as random effect. The number of days with diarrhea for each piglet during the 19 days following weaning was analyzed with management conditions and weaning age as main effects. The influence of management conditions and weaning age on the occurrence of diarrheic pigs (pigs expressing diarrhea at least once during the 19 days following weaning vs never) were analyzed using Khi2 test (*chisq*.*test* function).

Every piglet was then classified according to its expression of diarrhea (i.e. having exhibited diarrhea at least once or never during the 19 days following weaning for trial A, and at 33 or 47 days of age for trial B). For trial B only, piglets were also classified in two classes according to their ADG between 26 and 47 days of age and concentration of vitamin E at 26 days of age (lower or greater value than the median). Models including the class of diarrhea, growth or vitamin E, age (relatively to weaning in trial A or absolute age in trial B), and the corresponding interaction as main effects, and piglet as random effect were tested. For all analyses, when a significant effect (P < 0.05) was revealed by the analysis of variance, Tukey comparisons of adjusted means were performed using *cld* function from *lsmeans* package [17].

## Results

### Time-course of pro- and antioxidant blood variables between 12 and 147 days of age (trial A)

**Fig 1:**
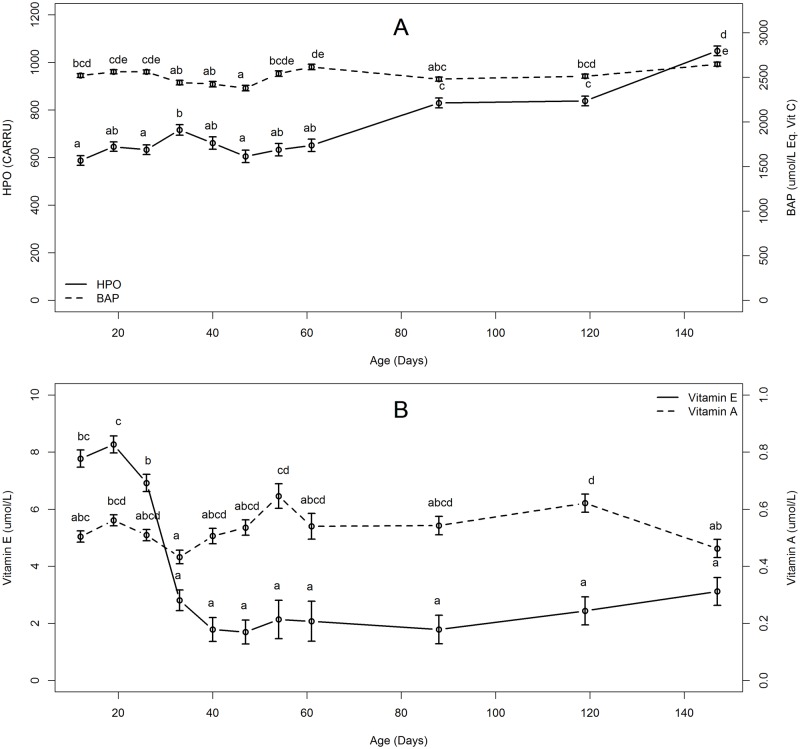
Plasma concentration of hydroperoxides (HPO), blood antioxidant capacity (BAP) (Fig 1A) and of vitamin E and A (Fig 1B) in pigs from 12 to 147 days of age (trial A). Half of the pigs were weaned at 21 days and half at 28 days of age. Least square means and standard error of mean (SEM) are shown. N = 64–66, excepted at 33 (n = 48) and between 40 and 61 days (n = 32). For each assay, means with different superscripts differ (P<0.05).

For this analysis, data from OC, DC, W21 and W28 piglets were pooled and analyzed for the age effect only. Plasma HPO increased transiently at 33 compared to 12 days of age (P < 0.001), dropped back to initial levels at 47 days of age, and then continuously increased until 147 days of age (P < 0.001, Fig 1A). Blood Antioxidant potential (BAP) decreased at 47 compared to 12 days of age (P < 0.01), came back to initial levels on day 54 and then remained stable until 147 days of age. Consequently, OSI followed HPO kinetic from 0.23 (± 0.008) at 12 days to 0.30 (± 0.008) CARRU.μmol^-1^.L of equivalent vitamin C at 33 days and increased until 0.40 (± 0.008) CARRU.μmol^-1^.L of eq vit C at 147 days of age.

Plasma concentration of vitamin E decreased from 19 to 40 days of age (respectively 8.26 vs 1.78 μmol/L, P < 0.001, Fig 1B) and then stayed stable until 147 days of age (3.12 μmol/L, P > 0.1, Fig 1B). Plasmatic vitamin A was stable between 12 and 147 days of age with however some variations and a transient decrease at 33 days of age (0.43 μmol/L) relatively to concentration at 12 days (P < 0.001) followed by an increase at 54 days of age (0.65 μmol/L, P < 0.001, Fig 1B).

### Influence of weaning age and management conditions on growth, diarrhea and on pro- and antioxidant blood variables (trial A)

Only data collected around weaning were included in this analysis. The statistical model included the effects of weaning condition (OC vs DC), age at weaning (W21 vs W28), and the time relatively to the day of weaning (from 9 days before to 19 days after). ADG was severely reduced after weaning, whatever age at weaning and management conditions compared to ADG during the lactation period (P < 0.001, Table 1). Between 5 and 11 days after weaning, ADG was lower for DC compared to OC piglets (P < 0.05), whereas it was similar for W21 and W28 piglets (P > 0.1, S1 Table).

**Table 1:** Blood oxidative status variables and growth from 9 days before to 19 days after weaning according to management conditions (optimal (OC, n = 34) or deteriorated (DC, n = 32), trial A).

		Time to weaning (days)						p-values^1^				
		d-9	d-2	d5	d12	d19	SEM	T	C	W	C*T	W*T
**HPO (CARRU)**	OC	621^a^	610^a^	679^a^^b^	663^a^^b^	592^a^	37	<0.001	0.337	0.340	0.054	0.030
	DC	620^a^	636^a^	658^a^	783^b^	668^a^^b^						
**BAP (μmol/L eq Vit C)**	OC	2547^b^^c^	2546^b^^c^	2543^b^^c^	2482^a^^b^^c^	2488^a^^b^^c^	46	<0.001	0.192	0.006	0.012	0.021
	DC	2546^c^	2569^c^	2473^b^^c^	2311^a^	2374^a^^b^						
**OSI (CARRU.μmol**^**-1**^**.L eq Vit C)**	OC	0.25^a^	0.24^a^	0.27^a^	0.28^a^	0.24^a^	0,02	<0.001	0.160	0.947	0.002	0.056
	DC	0.24^a^	0.25^a^	0.27^a^	0.35^b^	0.28^a^^b^						
**Vitamin E (μmol/L)**	OC	7.48^c^	8.24^c^^d^	4.12^b^	1.94^a^^b^	1.93^a^	0,60	<0.001	0.123	0.188	0.012	0.104
	DC	9.20^c^^d^	9.47^d^	3.52^a^^b^	2.01^a^^b^	1.61^a^						
**Vitamin A (μmol/L)**	OC	0.55	0.59	0.48	0.52	0.57	0,03	<0.001	0.006	0.391	0.305	0.761
	DC	0.53	0.53	0.4	0.38	0.52						
		**Birth to d-10**	**d-9 to d-3**	**d-2 to d4**	**d5 to d11**	**d12 to d18**						
**ADG (g/day)**	OC	228^b^^c^^d^	283^d^^e^	215^b^^c^	204^b^^c^	348^e^	17	<0.001	0.007	0.269	0.001	<0.001
	DC	236^b^^c^^d^	276^c^^d^^e^	175^b^	104^a^	276^c^^d^^e^						

^1^ P-value of the effects of time to weaning (T), management condition (C), weaning age (W) and their interactions (C*T and W*T) are presented.

^a-d^ For each variable, means with different superscripts differ (P<0.05).

HPO: hydroperoxides, BAP: blood antioxidant potential, OSI, Oxidative Stress Index (OSI = HPO/BAP), ADG: average daily gain

From 0 to 19 days after weaning, 55% of piglets exhibited at least one day of diarrhea (S2 Table). The duration of diarrhea varied between 1 and 6 days. The percentage of piglets which exhibited at least one day with diarrhea was not different between OC and DC (44% and 66% respectively, P > 0.1) and between W21 and W28 (66% and 44% respectively, P > 0.1). However, between 5 and 11 days after weaning, DC were more affected than OC piglets (60% vs 26% respectively, P < 0.05) and W21 more affected than W28 piglets (56% vs 30%, P < 0.05). Moreover, over the 0-19d period after weaning, DC piglets exhibited significantly more days with diarrhea compared to OC piglets (1.56 days +/- 1.64 vs 0.59 +/- 0.74, P < 0.01), and W21 piglets exhibited significantly more days with diarrhea compared to W28 piglets (1.44 days +/- 1.52 vs 0.70 +/- 1.06, P < 0.05).

Whatever weaning age, HPO increased and BAP decreased between -2 (d-2) and 12 days (d12) relatively to weaning in DC (P < 0.001and P < 0.01 respectively) but not OC piglets (P > 0.1, Table 1). Consequently, the OSI of DC piglets increased between d-2 and d12 (P < 0.01) resulting in a greater OSI than OC piglets on d12 (P < 0.05, Table 1). Whatever the weaning age and management conditions, plasma concentration of vitamin E dropped on d5 relatively to d-2 (P < 0.001), and then decreased until d19 (P < 0.001). Concentration of vitamin A decreased on d 5 (P < 0.001) and 12 (P < 0.001) compared to d-2, and was back to initial value on d19 (P > 0.1). BAP was greater at d5 for W21 compared to W28 (P < 0.05, 2595 vs 2422 μmol/L, S1 Table). HPO, OSI, vitamin E, and vitamin A concentrations were not affected by weaning age (P > 0.1, S1 Table).

Piglets which exhibited at least one day of diarrhea between 0 and 19 days after weaning had higher OSI on d12 compared to piglet which did not exhibit any diarrhea (P < 0.01, Table 2). BAP and HPO were not affected by the expression of diarrhea (P > 0.05). Vitamin A concentration was lower (P < 0.05) and vitamin E concentration tended to be greater (P < 0.1) in diarrheic pigs whatever the time relatively to weaning (Table 2).

**Table 2:** Blood oxidative status variables and growth in piglets exhibiting diarrheas (n = 36) or not (n = 30) between 0 and 19 days after weaning (trial A).

		Time to weaning (days)						p-values^1^		
		d-9	d-2	d5	d12	d19	SEM	T	D	T*D
**HPO (CARRU)**	No Diarrhea	645^a^	629^a^	656^a^^b^	642^a^^b^	627^a^	37	<0.001	0.707	0.011
	Diarrhea	601^a^	616^a^	682^a^^b^	786^b^	629^a^				
**BAP (μmol/L eq Vit C)**	No Diarrhea	2568	2561	2538	2441	2390	50	<0.001	0.452	0.285
	Diarrhea	2528	2552	2479	2356	2466				
**OSI (CARRU.μmol**^**-1**^**.L eq Vit C)**	No Diarrhea	0.25^a^^b^	0.25^a^^b^	0.26^a^^b^	0.26^a^^b^	0.27^a^^b^	0.02	<0.001	0.440	<0.001
	Diarrhea	0.24^a^	0.24^a^^b^	0.28^b^	0.35^c^	0.26^a^^b^				
**Vitamin E (μmol/L)**	No Diarrhea	7.25	8.52	3.66	2.03	1.64	0.63	<0.001	0.058	0.140
	Diarrhea	9.12	9.14	3.96	1.84	1.82				
**Vitamin A (μmol/L)**	No Diarrhea	0.57	0.59	0.47	0.49	0.55	0.03	<0.001	0.040	0.915
	Diarrhea	0.52	0.54	0.42	0.42	0.54				
		**Birth to d-10**	**d-9 to d-3**	**d-2 to d4**	**d5 to d11**	**d12 to d18**				
**ADG (g/day)**	No Diarrhea	246	295	213	192	325	19	<0.001	0.022	0.643
	Diarrhea	221	267	179	125	301				

^1^ P-value of the effects of time to weaning (T), Diarrhea (D) and their interactions (T*D) are presented.

^a-g^ For each variable, means with different superscripts differ (P<0.05)

HPO: hydroperoxides, BAP: blood antioxidant potential, OSI, Oxidative Stress Index (OSI = HPO/BAP), ADG: average daily gain

### Growth, diarrhea expression and pro- and antioxidant blood variables around weaning in commercial farms (trial B)

ADG was on average 190 g/day (from 111 to 281 g/day) during the first week around weaning (d26 to d33), 402 g/day (215 to 558 g/day) from d33 to d47 and 316 g/day (208 to 445 g/day) from d26 to d47. Because of the lack of attendance of farmers in recording sanitary events, diarrhea events could not be properly analyzed in this trial. During the farm visits at respectively 33 and 47 days of age, out of 288 piglets observed by experimenters, 21 and 27 piglets exhibited diarrhea. Piglets that exhibited diarrhea at 5 or 19 days after weaning (i.e. at 33 and 47 days of age) tended to display lower ADG compared to those which did not (respectively 177 vs 192 g/day at 33 days of age and 367 vs 407 g/day at 47 days of age, P = 0.09).

**Fig 2:**
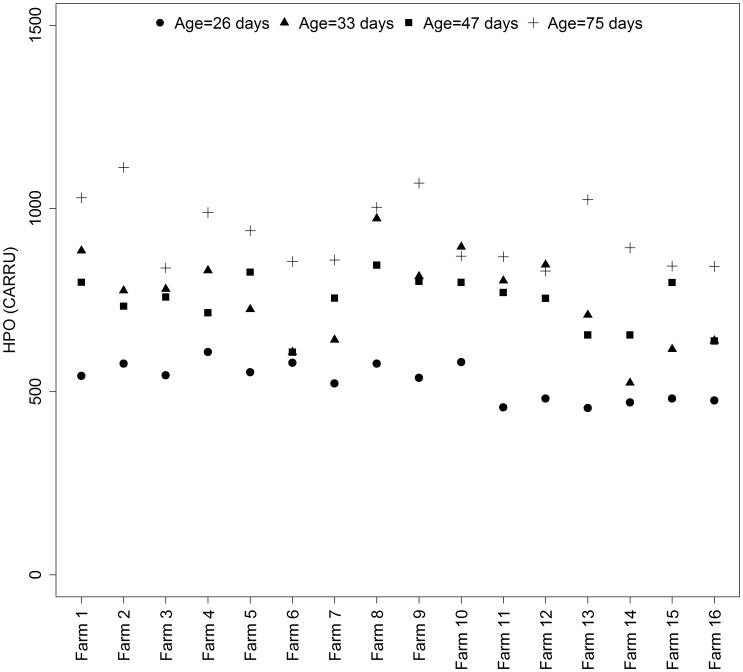
Mean plasma concentrations of hydroperoxides (HPO) in piglets aged from 26 to 75 days reared in 16 French commercial farms (trial B, n = 18 per farm).

Fig 2 illustrates the variability of HPO values according to piglet age in the 16 commercial farms enrolled in trial B. On average, HPO concentration increased from 528 to 754 CARRU between 26 and 33 days (P < 0.001) and reached maximal concentration at 75 days of age (927 CARRU, P < 0.001). Between 26 and 33 days of age, HPO concentrations increased (P < 0.05) in 14 farms and remained stable (P > 0.1) in 2 farms. Between 33 and 47 days of age, they remained stable (P > 0.1) in 12 farms, decreased in 1 farm (P<0.05) and increased (P < 0.05) in 3 farms. Between 47 and 75 days of age, they increased (P<0.05) in 8 farms and remained stable (P > 0.1) in 8 farms.

BAP increased transiently from 2255 to 2493 μmol/L of eq vit C between 26 and 47 days of age (P < 0.001) and then decreased to 2361 μmol/L of eq vit C at 75 days of age (P < 0.001, Table 3). OSI increased between 26 and 33 days of age (from 0.23 to 0.34 CARRU/ μmol/L of eq vit C, P < 0.001), decreased at 47 days of age and then increased at 75 days of age (0.30 and 0.39 CARRU/ μmol/L of eq vit C, P < 0.001). Concentrations in vitamin E (7.84 to 3.59 μmol/L) and A (0.52 to 0.45 μmol/L) decreased between 26 and 33 days of age (P < 0.001, Table 3). Whatever the age, piglets displaying low ADG between 26 and 47 days of age ([-109; 317] g/day) had lower BAP compared to piglets with high ADG ([318; 709] g/day, P < 0.001, Table 3). Low ADG piglets had greater HPO concentration at 33 days of age (P < 0.01) and OSI at 33 (P < 0.001) and 47 days of age (P < 0.01). Vitamin A concentrations were similar between the two groups of piglets at 26 days of age, but they were decreased at 33 days of age in low ADG piglets (P < 0.001). Interestingly, low ADG piglets had lower pre-weaning concentrations in vitamin E (P < 0.05). Consequently, we then compared piglets with low and high concentration of vitamin E at 26 days of age. Piglets with the highest concentration ([7.80; 21.1] μmol/L) weighed 8.27 ± 0.48 kg at 26 days of age, which was not different from the weight of piglets with the lowest vitamin E concentration ([0.17; 7.79] μmol/L, 8.18 ± 0.48kg, P > 0.1). However, high vitamin E pigs had a better growth between 33 and 47 days of age (420 vs 382 g/day, P < 0.001) resulting in a higher weight at 47 days of age (15.5 vs 14.6 kg, P < 0.001).

**Table 3:** Blood oxidative status variables and growth in piglets from the commercial farms displaying lower (“low ADG”, -109 to 317 g/day, n = 142) or greater (“high ADG”, 318 to 709 g/day, n = 140) ADG than the median between 26 and 47 days of age (trial B).

		Age (Days)					p-value		
	Growth	26	33	47	75	SEM	A	G	A*G
**HPO (CARRU)**	low ADG	528^a^	792^c^	763^b^^c^	910^d^	15	<0.001	0.123	<0.001
	high ADG	528^a^	719^b^	723^b^	945^d^				
**BAP (μmol/L eq Vit C)**	low ADG	2237	2262	2440	2338	21	<0.001	<0.001	0.218
	high ADG	2270	2326	2547	2383				
**OSI (CARRU.μmol**^**-1**^**.L eq Vit C)**	low ADG	0.24^a^	0.36^d^	0.32^c^	0.39^e^	0.01	<0.001	0.001	<0.001
	high ADG	0.23^a^	0.31^c^	0.28^b^	0.40^e^				
**Vitamin E (μmol/L)**	low ADG	7.17^b^	3.27^a^			0.21	<0.001	<0.001	0.055
	high ADG	8.55^c^	3.90^a^						
**Vitamin A (μmol/L)**	low ADG	0.51^b^	0.40^a^			0.01	<0.001	<0.001	0.001
	high ADG	0.53^b^	0.51^b^						
			**26 to 33**	**33 to 47**					
**ADG (g/day)**	low ADG		209^b^	450^d^		10	<0.001	<0.001	<0.001
	high ADG		170^a^	352^c^					

^1^ P-value of the effects of Age (A), Growth (G) and their interactions (A*G) are presented.

^a-e^ For each variable, means with different superscripts differ (P<0.05)

HPO: hydroperoxides, BAP: blood antioxidant potential, OSI, Oxidative Stress Index (OSI = HPO/BAP), ADG: average daily gain

## Discussion

To our knowledge, this is the first study exploring oxidative status in young mammals from neonatal period to puberty. Indeed, on mammals, the evolution of oxidative products has been mainly explored just after birth or at senescence [1,18]. At birth, neonate moves from uterus to an external environment richer in oxygen leading to oxidative burst [18]. With senescence, oxidative damages caused to macromolecules (lipids, DNA and proteins) increase compared to mature adults, which leads to the loss of cellular functions [1]. This phenomenon has been shown in old adults for several species (human: [19], rabbit: [20], hare: [21], sheep: [22]). Results of trial A show that the concentration of oxidative products increases continuously with age in young pigs. This increase occurred while the plasma antioxidant potential as well as vitamin A concentrations remained stable. Similarly, with the exception of the initial drop due to weaning, vitamin E remained stable until puberty. Blood is the place where oxidative products from diverse origins accumulate. The increase in hydroperoxides with age in young developing animals might reflect a high rate of reactive oxygen species production resulting from the high cellular activity for tissue accretion. The possible pro-oxidant effect of growth is supported by the observation that fast growing lines of pigs have deteriorated redox status compared to slow growing lines [23,24].

At weaning, in trial A, plasma concentration of HPO increased strongly whereas antioxidant capacity decreased resulting in an increase in OSI, at least for piglets weaned in deteriorated conditions. The phenomenon of oxidative stress after weaning has been substantially described in piglets weaned between 21 and 28 days of age [7–10,25]. However, those studies did not demonstrate that the observed oxidative stress was due to weaning and not to natural ageing. In the present study, DC piglets weaned at 21 and 28 days of age displayed comparable patterns of variations in HPO, BAP, OSI and vitamins A and E the days following weaning, confirming that the observed effects are effectively linked to weaning and not to a physiological process evolving only with age. However, the comparison of optimal and deteriorated weaning conditions showed that the occurrence of oxidative stress depends on the management and environment at weaning. High animal density, animal mixing, lack of cleaning and disinfection of the room as well as thermic stress are known to be risks factors of health problems and deteriorated growth [11]. In the experimental model developed in trial A, deteriorated conditions included several of these factors. On field conditions, in trial B, the range of HPO increase and the delay before recovery to pre-weaning concentrations seemed to be greater than in experimental facilities in most of the farms. Indeed, HPO concentrations were still higher than pre-weaning level 19 days after weaning in 14 farms out of 16. This might be due to more challenging rearing and weaning conditions in commercial farms than in our experimental facilities. Trial A results show that animals optimally managed at weaning can contain their oxidative stress.

In non-optimal weaning conditions, different causes can cumulate to generate oxidative stress. At weaning, separation from the mother and adaptation to a new physical and social environment generate a stress response, with an increase of plasma cortisol concentration, an hormone secreted by the hypothalamic-pituitary-adrenal axis [26]. Studies on growing chicken showed that glucocorticoids, added in the feed [27] or induced by thermic stress [28], increased the production of oxidative products. Thus, the neuroendocrine stress response to weaning could contribute to oxidative stress at that period.

Weaning also induces a systemic and local inflammation, as shown for example by the increase in plasma concentration of a pro-inflammatory cytokine, interleukin-1, one to two days after weaning [29] or in the haptoglobin acute phase protein 5 days after weaning [25]. At the gut level, local inflammatory responses are observed directly after weaning [30], leading in extreme cases to the exhibition of diarrhea [31]. Since inflammation is known to increase oxidative stress on farm animals [3,32,33], we can hypothesize that oxidative stress at weaning is also partly induced by inflammation.

In trial A, DC conditions were used to increase the stress of the animals (social stress) and challenge their immune system (cold stress, poor hygiene). The social stress of the animals was not measured, but the greater number of DC piglets with diarrheas for a longer period of time, compared to OC pigs suggests that the model was effective in decreasing piglet health at weaning. Their poorer health was also attested by their more severe degradation of ADG after weaning compared to OC pigs. Thus the DC model efficiently contributed to health degradation undergone by piglets at weaning and this was associated to more oxidative stress as indicated by a greater OSI 12 days after weaning. Exhibition of diarrhea in trial B was not associated with the evolution of ADG and oxidative parameters, maybe because the majority of diarrhea events were probably missed due to the deficient recording of individual sanitary events in this field study. Nevertheless, data from trial B, also suggest a link between oxidative status and health at weaning. Indeed, piglets with the lowest ADG had significantly more oxidative products, greater OSI and lower vitamin A concentration after weaning compared to piglets with the highest growth.

Usually, the increase in free radical production by the organism consumes neutralizing antioxidant molecules, and enhances the production of antioxidant enzymes and molecules [1,2]. In trial A, blood antioxidant capacity was transiently decreased 12 days after weaning, which probably reflects the use of antioxidant reserves shown, in our study, by the decrease in vitamin E and to a less extent in vitamin A plasma concentrations. The dramatic drop of vitamin E after weaning was reported in several other studies and the absolute values as well as the amplitude of the decrease observed in both trials are in accordance with the literature [34–37]. The piglet, like other mammals, is unable to synthetize vitamin E which has to be provided by the diet [38]. Lipid soluble vitamins E and A are absorbed by the intestine partly thanks to the action of lipases [39] and bile acids [40], whose activity is reduced after weaning [41], thus reducing vitamin A and E absorption. Furthermore, concentration of total lipids in the diet influences the absorption of vitamin E by the intestine [35,39]. Milk contains more fat content than starter feed [42] and thus vitamin E absorption is presumed to be higher before weaning. Consequently, the high consumption of vitamin E for pro-oxidant molecule neutralization as well as the reduction of vitamin E supply to the organism could explain the drop of vitamin E after weaning. Interestingly, piglets that displayed the lowest ADG between 26 and 47 days of age, had lower plasma concentration of vitamin E but the same weight just before weaning, as well as greater HPO concentrations 5 days after weaning. This reinforces the hypothesis of a major role of vitamin E stores before weaning.

Absolute values observed for concentration in HPO before weaning (532 CARRU at 26 days of age in average for trial A and B together) are greater than values observed on piglets before weaning in other studies: 495 CARRU at 20 days old [9], 366 CARRU at 23 days of age [9], 400 CARRU at 24 days of age [8] or 350 CARRU at 28 days of age [7]. Currently, no reference values of concentrations of HPO on piglets before weaning are available. The lack of references for oxidative status, and particularly HPO as health indicator and to qualify situation of oxidative stress, has been already underlined for ruminants [43]. Our data showed that variability within and between farms is quite important. Consequently, the relative increase between pre and post-weaning values could be a better indicator for the between farm comparison.

## Conclusions

Oxidative status of pig continuously evolves from neonatal period to puberty because of the increase in plasma concentration of HPO and the stabilization of antioxidant capacity (BAP). Weaning induces a transient oxidative stress associated with diarrhea and that could be limited by optimal management conditions. OSI followed ADG and diarrhea expression, and thus could be suggested as a good indicator of piglet health at weaning. The plasma concentration of vitamin E before weaning is associated with growth. The causal relationship between oxidative stress, vitamin E stores, and health after weaning needs to be explored in order to find ways to contain oxidative stress and maintain health after weaning.

## Supporting information

S1 File Raw dataset of trial A and trial B. (XLSX)

S1 Table Blood oxidative status variables and growth from 9 days before to 19 days after weaning according to age at weaning (21 days of age (W21, n = 32) or 28 days of age (W28, n = 34), trial A). (DOCX)

S2 Table Expression of diarrhea according to the treatment groups of piglets (trial A): piglets weaned at 21 (W21, n = 32) or 28 days of age (W28, n = 34) and housed in optimal (OC, n = 34) or deteriorated conditions (DC, n = 32). (DOCX)

## References

[1] Halliwell B, Gutteridge JM. Free radicals in biology and medicine. Oxford University Press, USA; 2015.

[2] Kohen R, Nyska A. Oxidation of Biological Systems: Oxidative Stress Phenomena, Antioxidants, Redox Reactions, and Methods for Their Quantification. Toxicol Pathol. 2002;30: 620–650. 10.1080/01926230290166724 12512863

[3] Lykkesfeldt J, Svendsen O. Oxidants and antioxidants in disease: Oxidative stress in farm animals. Vet J. 2007;173: 502–511. 10.1016/j.tvjl.2006.06.005 16914330

[4] Funderburke DW, Seerley RW. The effects of postweaning stressors on pig weight change, blood, liver and digestive tract characteristics. J Anim Sci. 1990;68: 155–162. 2303393 10.2527/1990.681155x

[5] Pluske JR, Le Dividich J, Verstegen MWA. Weaning the pig: concepts and consequences. Wageningen: Wageningen Academic Publishers; 2003.

[6] Weary DM, Jasper J, Hötzel MJ. Understanding weaning distress. Early Weaning. 2008;110: 24–41. 10.1016/j.applanim.2007.03.025

[7] Corino C, Rossi R, Musella M, Cannata S, Pastorelli G. Growth performance and oxidative status in piglets supplemented with verbascoside and teupolioside. Ital J Anim Sci. 2007;6: 292–294.

[8] Pastorelli G, Rossi R, Corino C. Influence of Lippia citriodora verbascoside on growth performance, antioxidant status, and serum immunoglobulins content in piglets. Czech J Anim Sci. 2012;57: 312–322.

[9] Sauerwein H, Schmitz S, Hiss S. Effects of a dietary application of a yeast cell wall extract on innate and acquired immunity, on oxidative status and growth performance in weanling piglets and on the ileal epithelium in fattened pigs. J Anim Physiol Anim Nutr. 2007;91: 369–380. 10.1111/j.1439-0396.2006.00663.x 17845244

[10] Zhu LH, Zhao KL, Chen XL, Xu JX. Impact of weaning and an antioxidant blend on intestinal barrier function and antioxidant status in pigs. J Anim Sci. 2012;90: 2581–2589. 10.2527/jas.2012-4444 22896732

[11] Madec F, Bridoux N, Bounaix S, Jestin A. Measurement of digestive disorders in the piglet at weaning and related risk factors. Prev Vet Med. 1998;35: 53–72. 10.1016/S0167-5877(97)00057-3 9638780

[12] Alberti A, Bolognini L, Macciantelli D, Caratelli M. The radical cation of N,N-diethyl-para-phenylendiamine: A possible indicator of oxidative stress in biological samples. Res Chem Intermed. 1999;26: 253–267. 10.1163/156856700X00769

[13] Benzie IFF, Strain JJ. The Ferric Reducing Ability of Plasma (FRAP) as a Measure of “Antioxidant Power”: The FRAP Assay. Anal Biochem. 1996;239: 70–76. 10.1006/abio.1996.0292 8660627

[14] Sharma RK, Pasqualotto FF, Nelson DR, Thomas AJ, Agarwal A. The reactive oxygen species—total antioxidant capacity score is a new measure of oxidative stress to predict male infertility. Hum Reprod. 1999;14: 2801–2807. 10.1093/humrep/14.11.2801 10548626

[15] Bates D, Mächler M, Bolker B, Walker S. Fitting linear mixed-effects models using lme4. 2014;

[16] R Core Team. R: A Language and Environment for Statistical Computing. Vienna, Austria: R Foundation for Statistical Computing; 2015.

[17] Lenth R. lsmeans: Least-Squares Means. 2015.

[18] Mutinati M, Pantaleo M, Roncetti M, Piccinno M, Rizzo A, Sciorsci R. Oxidative stress in neonatology. A review. Reprod Domest Anim. 2014;49: 7–16. 10.1111/rda.12230 24112309

[19] Finkel T, Holbrook NJ. Oxidants, oxidative stress and the biology of ageing. Nature. 2000;408: 239–247. 10.1038/35041687 11089981

[20] Oriani G, Corino C, Pastorelli G, Pantaleo L, Ritieni A, Salvatori G. Oxidative status of plasma and muscle in rabbits supplemented with dietary vitamin E. J Nutr Biochem. 2001;12: 138–143. 10.1016/S0955-2863(00)00132-7 11257462

[21] Palazzo M, Vizzarri F, Cinone M, Corino C, Casamassima D. Assessment of a natural dietary extract, titrated in phenylpropanoid glycosides, on blood parameters and plasma oxidative status in intensively reared Italian hares (Lepus corsicanus). Animal. 2011;5: 844–850. 10.1017/S1751731110002569 22440023

[22] Casamassima D, Palazzo M, D’alessandro A, Colella G, Vizzarri F, Corino C. The effects of lemon verbena (Lippia citriodora) verbascoside on the productive performance, plasma oxidative status, and some blood metabolites in suckling lambs. J Anim Feed Sci. 2013;22: 204–212.

[23] Brambilla G, Civitareale C, Ballerini A, Fiori M, Amadori M, Archetti LI, et al Response to oxidative stress as a welfare parameter in swine. Redox Rep. 2002;7: 159–163. 10.1179/135100002125000406 12189046

[24] Merlot E, Vincent A, Thomas F, Meunier-Salaün M-C, Damon M, Robert F, et al Health and immune traits of Basque and Large White pigs housed in a conventional or enriched environment. Animal. 2012;6: 1290–1299. 10.1017/S1751731112000080 23217232

[25] Sauerwein H, Schmitz S, Hiss S. The acute phase protein haptoglobin and its relation to oxidative status in piglets undergoing weaning-induced stress. Redox Rep. 2005;10: 295–302. 10.1179/135100005X83725 16438801

[26] Colson V, Martin E, Orgeur P, Prunier A. Influence of housing and social changes on growth, behaviour and cortisol in piglets at weaning. Tufts Univ Spec Sect. 2012;107: 59–64. 10.1016/j.physbeh.2012.06.001 22691708

[27] Lin H, Decuypere E, Buyse J. Oxidative stress induced by corticosterone administration in broiler chickens (Gallus gallus domesticus): 1. Chronic exposure. Comp Biochem Physiol B Biochem Mol Biol. 2004;139: 737–744. 10.1016/j.cbpc.2004.09.013 15581806

[28] Akbarian A, Michiels J, Degroote J, Majdeddin M, Golian A, De Smet S. Association between heat stress and oxidative stress in poultry; mitochondrial dysfunction and dietary interventions with phytochemicals. J Anim Sci Biotechnol. 2016;7: 1–14. 27354915 10.1186/s40104-016-0097-5 PMC4924307

[29] McCracken BA, Gaskins HR, Ruwe-Kaiser PJ, Klasing KC, Jewell DE. Diet-Dependent and Diet-Independent Metabolic Responses Underlie Growth Stasis of Pigs at Weaning. J Nutr. 1995;125: 2838–2845. 7472664 10.1093/jn/125.11.2838

[30] McCracken BA, Spurlock ME, Roos MA, Zuckermann FA, Gaskins HR. Weaning Anorexia May Contribute to Local Inflammation in the Piglet Small Intestine. J Nutr. 1999;129: 613–619. 10082764 10.1093/jn/129.3.613

[31] Fairbrother JM, Nadeau É, Gyles CL. Escherichia coli in postweaning diarrhea in pigs: an update on bacterial types, pathogenesis, and prevention strategies. Anim Health Res Rev. 2005;6: 17–39. 10.1079/AHR2005105 16164007

[32] Lauritzen B, Lykkesfeldt J, Friis C. Evaluation of a single dose versus a divided dose regimen of amoxycillin in treatment of Actinobacillus pleuropneumoniae infection in pigs. Res Vet Sci. 2005;79: 61–67. 10.1016/j.rvsc.2004.09.011 15894026

[33] Royer E, Barbe F, Rousselière Y, Chevaux E, Guillou D. Développement d’un modèle d’étude du stress oxydant chez le porcelet sevré. Journ Rech Porc. 2016;48: 341–346.

[34] Lauridsen C, Jensen SK. Influence of supplementation of all-rac-α-tocopheryl acetate preweaning and vitamin C postweaning on α-tocopherol and immune responses of piglets. J Anim Sci. 2005;83: 1274–1286. 15890805 10.2527/2005.8361274x

[35] Moreira I, Mahan DC. Effect of dietary levels of vitamin E (all-rac-tocopheryl acetate) with or without added fat on weanling pig performance and tissue alpha-tocopherol concentration. J Anim Sci. 2002;80. 10.2527/2002.803663x 11890402

[36] Sivertsen T, Vie E, Bernhoft A, Baustad B. Vitamin E and selenium plasma concentrations in weanling pigs under field conditions in Norwegian pig herds. Acta Vet Scand. 2007;49: 1–1. 10.1186/1751-0147-49-1 17201915 PMC1779789

[37] Wilburn EE, Mahan DC, Hill DA, Shipp TE, Yang H. An evaluation of natural (RRR-α-tocopheryl acetate) and synthetic (all-rac-α-tocopheryl acetate) vitamin E fortification in the diet or drinking water of weanling pigs123. J Anim Sci. 2008;86. 10.2527/jas.2007-0377 18156353

[38] Traber MG. Vitamin E In: Ross AC, Caballero B, Cousins RJ, Tucker KL, Ziegler TR, editors. Modern Nutrition in Health and Disease. 11th ed Baltimore: Lippincott Williams & Wilkins; 2012.

[39] Kayden HJ, Traber MG. Absorption, lipoprotein transport, and regulation of plasma concentrations of vitamin E in humans. J Lipid Res. 1993;34: 343–358. 8468520

[40] Lauridsen C, Hedemann MS, Jensen SK. Hydrolysis of tocopheryl and retinyl esters by porcine carboxyl ester hydrolase is affected by their carboxylate moiety and bile acids. J Nutr Biochem. 2001;12: 219–224. 10.1016/S0955-2863(00)00156-X 11287217

[41] Jensen MS, Jensen SK, Jakobsen K. Development of digestive enzymes in pigs with emphasis on lipolytic activity in the stomach and pancreas. J Anim Sci. 1997;75: 437–445. 10.2527/1997.752437x 9051467

[42] Bark LJ, Crenshaw TD, Leibbrandt VD. The Effect of Meal Intervals and Weaning on Feed Intake of Early Weaned Pigs. J Anim Sci. 1986;62: 1233–1239. 3722016 10.2527/jas1986.6251233x

[43] Celi P. Biomarkers of oxidative stress in ruminant medicine. Immunopharmacol Immunotoxicol. 2010;33: 233–240. 10.3109/08923973.2010.514917 20849293

