# Supplementary material for: Effects of age and weaning conditions on blood indicators of oxidative status in pigs
Source: PLoS One. 2017 May 24;12(5):e0178487. doi: 10.1371/journal.pone.0178487 (PMC5443573; doi:10.1371/journal.pone.0178487)
Supplement: S1 Table — (DOCX) [file pone.0178487.s002.docx]

**S1 Table:** Blood oxidative status variables and growth from 9 days before to 19 days after weaning according to age at weaning (21 days of age (W21, n=32) or 28 days of age (W28, n=34), trial A)

|  |  | **Time to weaning (days)** | | | | |  | **p-values^1^** | | | | |
| --- | --- | --- | --- | --- | --- | --- | --- | --- | --- | --- | --- | --- |
|  |  | **d-9** | **d-2** | **d5** | **d12** | **d19** | **SEM** | **T** | **C** | **W** | **C*T** | **W*T** |
| **HPO (CARRU)** | W21 | 600^a^ | 651^a^ | 673^ab^ | 780^b^ | 654^ab^ | 37 | <0.001 | 0.337 | 0.340 | 0.054 | 0.030 |
|  | W28 | 641^ab^ | 596^a^ | 664^ab^ | 666^ab^ | 656^a^ |  |  |  |  |  |  |
| **BAP (µmol/L eq Vit C)** | W21 | 2556^bc^ | 2587^c^ | 2595^c^ | 2452^abc^ | 2512^abc^ | 46 | <0.001 | 0.192 | 0.006 | 0.012 | 0.021 |
|  | W28 | 2537^c^ | 2528^bc^ | 2422^ab^ | 2341^a^ | 2350^a^ |  |  |  |  |  |  |
| **OSI (CARRU.µmol^-1^.L eq Vit C)** | W21 | 0.23 | 0.25 | 0.26 | 0.33 | 0.26 | 0.02 | <0.001 | 0.160 | 0.947 | 0.002 | 0.056 |
|  | W28 | 0.25 | 0.24 | 0.28 | 0.29 | 0.26 |  |  |  |  |  |  |
| **Vitamin E (µmol/L)** | W21 | 8.63 | 8.56 | 4.58 | 2.27 | 1.97 | 0.6 | <0.001 | 0.123 | 0.188 | 0.012 | 0.104 |
|  | W28 | 8.05 | 9.15 | 3.06 | 1.68 | 1.56 |  |  |  |  |  |  |
| **Vitamin A (µmol/L)** | W21 | 0.55 | 0.59 | 0.48 | 0.52 | 0.57 | 0.03 | <0.001 | 0.006 | 0.391 | 0.305 | 0.761 |
|  | W28 | 0.53 | 0.53 | 0.40 | 0.38 | 0.52 |  |  |  |  |  |  |
|  |  |  |  |  |  |  |  |  |  |  |  |  |
|  |  | **Birth to d-10** | **d-9 to d-3** | **d-2 to d4** | **d5 to d11** | **d12 to d18** |  |  |  |  |  |  |
| **ADG (g/day)** | W21 | 200^bc^ | 270^de^ | 226^bcd^ | 119^a^ | 315^e^ | 18 | <0.001 | 0.007 | 0.269 | 0.001 | <0.001 |
|  | W28 | 264^cde^ | 289^de^ | 164^ab^ | 189^ab^ | 309^e^ |  |  |  |  |  |  |

^1^ P-value of the effects of time to weaning (T), management condition (C), weaning age (W) and their interactions (C*T and W*T) are presented.

^a-d^ For each variable, means with different superscripts differ (P<0.05).

HPO: hydroperoxides, BAP: blood antioxidant potential, OSI, Oxidative Stress Index (OSI = HPO/BAP), ADG: average daily gain
