# Supplementary material for: Effects of age and weaning conditions on blood indicators of oxidative status in pigs
Source: PLoS One. 2017 May 24;12(5):e0178487. doi: 10.1371/journal.pone.0178487 (PMC5443573; doi:10.1371/journal.pone.0178487)
Supplement: S2 Table — (DOCX) [file pone.0178487.s003.docx]

**S2 Table:** Expression of diarrhea according to the treatment groups of piglets (trial A): piglets weaned at 21 (W21, n=32) or 28 days of age (W28, n=34) and housed in optimal (OC, n=34) or deteriorated conditions (DC, n=32).

|  | **W21-DC** | **W21-OC** | **W28-DC** | **W28-OC** | **Significant effects^1^** |
| --- | --- | --- | --- | --- | --- |
| **% of piglet exhibiting diarrhea at least once during the 19 days after weaning:** | | | | | |
|  | 69 | 63 | 63 | 28 | - |
| **% of piglet exhibiting diarrhea at least once between 5 and 11 days after weaning:** | | | | | |
|  | 69 | 44 | 50 | 11 | C, W |
| **Number of days of diarrhea expression per pig during the 0-19 day post-weaning period:** | | | | | |
|  | 2,1 | 0,8 | 1,1 | 0,4 | C, W |

^1^ C or W indicates significant (P < 0.05) condition or weaning age effects respectively.
